# Enfortumab vedotin promotes PD-L1 expression in urothelial carcinoma via NF-κB and STAT3 pathways highlighting mechanisms of immune evasion and potential for combination therapy

**DOI:** 10.1186/s12865-025-00751-2

**Published:** 2025-09-25

**Authors:** Hirohito Naito, Rikiya Taoka, Xia Zhang, Akram Hossain, Yohei Abe, Mikio Sugimoto

**Affiliations:** Department of Urology, Faculty of Medicine, Kagawa University, 1750-1, Ikenobe, Miki-cho, Kita-gun, 761-0793 Kagawa, Japan

**Keywords:** Urothelial carcinoma, Enfortumab vedotin, PD-1/PD-L1 inhibitor, NF-κB, STAT3

## Abstract

**Background:**

PD-1/PD-L1 inhibitors have revolutionized urothelial carcinoma (UC) treatment; however, the effects of prior or concurrent therapies on PD-L1 regulation remain unclear. This study investigates whether enfortumab vedotin (EV), a nectin-4-targeting antibody-drug conjugate, modulates PD-L1 expression in UC cells, and explores the underlying molecular pathways.

**Methods:**

UC cell lines RT4 (high nectin-4 expression) and T24 (low nectin-4 expression) were treated with EV (10 µg/ml) for 6, 12, or 24 h, followed by a 48-hour drug-free period. Protein and mRNA expression levels of PD-L1, NF-κB, and STAT3 were quantified using western blotting and qRT-PCR.

**Results:**

EV treatment upregulated PD-L1, NF-κB, and STAT3 in a time-dependent manner, with a more pronounced effect observed in RT4 than in T24 cells. PD-L1 protein levels increased 0.761-fold (12 h) and 2.399-fold (24 h) in RT4, whereas T24 showed a decrease (0.517-fold at 12 h) or minimal change (0.006-fold at 24 h). NF-κB expression increased 64.42-fold (12 h) and 97.03-fold (24 h) in RT4, compared to 1.251-fold (12 h) and 1.210-fold (24 h) in T24. STAT3 levels rose 2.334-fold (12 h) and 2.844-fold (24 h) in RT4, whereas T24 showed increases of 1.620-fold (12 h) and 1.379-fold (24 h). At the mRNA level (6 h post-treatment), PD-L1, NF-κB, and STAT3 were upregulated by 1.228-, 1.332-, and 1.225-fold, respectively, in RT4 cells.

**Conclusion:**

EV is associated with increased PD-L1 expression, along with upregulation of NF-κB and STAT3, suggesting a mechanistic link that may contribute to immune modulation in nectin-4-high bladder cancer cells. These findings highlight the need for combination strategies integrating EV with PD-1/PD-L1 inhibitors to optimize therapeutic outcomes in UC.

**Supplementary Information:**

The online version contains supplementary material available at 10.1186/s12865-025-00751-2.

## Introduction

Urothelial carcinoma (UC) is diagnosed in nearly 600,000 new cases annually and is responsible for over 200,000 deaths worldwide, making it the ninth most common malignant neoplasm [1]. UC is the predominant histologic subtype of bladder cancer, accounting for 90% of bladder cancer cases [2]. and 7% of all kidney cancer cases, including malignancies in the renal pelvis and ureter. The prognosis for metastatic bladder cancer remains particularly poor, with a five-year survival rate of only 5–6% [3]. Platinum-based chemotherapy is the standard first-line treatment for advanced UC; however, response rates remain low (8–30%), and survival after platinum-based chemotherapy failure is limited to 7–10 months [4]. To improve outcomes, maintenance therapy with avelumab, a programmed death-ligand 1 (PD-L1) inhibitor, has been introduced for patients without disease progression following front-line platinum-based chemotherapy [5]. Additionally, pembrolizumab, a programmed cell death protein 1 (PD-1) inhibitor, has shown efficacy for use in second-line treatment [6].

Enfortumab vedotin (EV), an antibody-drug conjugate targeting nectin-4, has significantly improved survival in patients with locally advanced or metastatic UC who previously received platinum-based chemotherapy and a PD-1/PD-L1 inhibitor [7]. EV consists of a fully human monoclonal antibody specific to nectin-4, conjugated to monomethyl auristatin E (MMAE), a microtubule-disrupting agent. Recently, the phase III EV-302 trial evaluated EV in combination with pembrolizumab in previously untreated patients with advanced UC, demonstrating superior clinical benefits and a manageable safety profile compared to chemotherapy. The median overall survival was 31.5 months in the group that received combination therapy with EV and pembrolizumab, and 16.1 months in the chemotherapy group [8]. Based on these results, the combination therapy is recommended as first-line treatment for metastatic or advanced urothelial carcinoma in many guidelines.

Overall survival was significantly prolonged in the combination therapy group. PD-L1 expression has emerged as a potential predictive biomarker for PD-1/PD-L1 inhibitor efficacy, with improved outcomes observed in tumors wherein the PD-L1 combined positive score exceeds 1% [8].

Notably, chemotherapy can influence PD-L1 expression in cancer cells, potentially affecting subsequent treatment strategies [9]. Given the evolving landscape of immunotherapy, understanding how prior or concurrent treatments impact PD-1/PD-L1 pathways is essential for optimizing treatment sequencing. However, evidence in this area remains limited. Recent studies have highlighted chemotherapy-induced upregulation of PD-L1. For example, Tsai et al. reported that cisplatin enhances PD-L1 but not PD-L2 expression in UC cell lines, as shown using western blotting and quantitative real-time PCR [9]. While similar findings were observed in head and neck squamous cell carcinoma cell lines treated with cisplatin [10], paclitaxel treatment increased PD-L1 expression in a mouse model of ovarian cancer [11]. These findings suggest that chemotherapy-induced modulation of PD-L1 expression may influence the efficacy of subsequent PD-1/PD-L1-targeted therapies in UC.

As MMAE, a component of EV, is a microtubule inhibitor similar to paclitaxel, it is plausible that EV may also regulate PD-L1 expression. However, this possibility has not yet been explored. This study aims to investigate the effects of EV treatment on PD-L1 expression in human UC cell lines and elucidate the underlying molecular mechanisms, providing insights into its potential impact on immunotherapy strategies.

## Materials and methods

### Cell culture

The human urothelial carcinoma (UC) cell lines RT4 and T24 were obtained from the Japan Cancer Research Bank (Tokyo, Japan). These cells were cultured in RPMI-1640 medium (Wako, Osaka, Japan) supplemented with 10% fetal bovine serum (FBS; Sigma-Aldrich, St. Louis, MO, USA), 2% HEPES solution (Sigma-Aldrich), and 1% penicillin-streptomycin (Thermo Fisher Scientific, Waltham, MA, USA). Cultures were maintained at 37 °C in a Humidified incubator with 5% CO₂.

### Reagents

EV was provided by Astellas Pharma Inc. (Tokyo, Japan) as part of a research project. Goat anti-rabbit IgG-conjugated horseradish peroxidase (HRP; cat. no. ab6721) and rabbit monoclonal antibody against STAT3 (cat. no. ab68153) were purchased from Abcam (Cambridge, UK). Rabbit polyclonal antibody against Nectin-4/PVRL4 (cat. no. 17402) and rabbit monoclonal antibodies against PD-L1 (cat. no. 13684) were purchased from Cell Signaling Technology (Danvers, MA, U.S.A.). Rabbit polyclonal antibody against NF-kB p65 (cat. no.AF0874) was purchased from Affinity Biosciences (Jiangsu, China). Rabbit polyclonal antibody against GAPDH (cat. no. sc-25778) was purchased from Santa Cruz Biotechnology (Dallas, Texas, U.S.A.).

### Cell viability assay

Cell viability was evaluated using the 3-(4,5-dimethylthiazol-2-yl)−2,5-diphenyltetrazolium bromide (MTT) assay. RT4 and T24 cells were seeded into 96-well plates at a density of 5 × 10³ cells/well and incubated for 24 h. The cells were then treated with EV at a concentration of 10–25 µg/mL for 12–24 h.

Following EV treatment, the cells were further incubated without EV for 12, 24, or 48 h. Control cells received an equivalent volume of RPMI-1640 medium instead of EV. Cell viability was assessed using the Cell Proliferation Kit I (Roche, Mannheim, Germany). Absorbance was measured at 570 nm, with 750 nm as the reference wavelength. All experiments were performed in triplicate or more to ensure reproducibility.

### Western blot analysis

RT4 and T24 cells were seeded into 10 cm dish at a density of 5 × 10⁴ cells/well and incubated for 24 h. The cells were then treated with EV at 10 µg/mL for 12–24 h, followed by an additional 48-hour incubation without EV. Both attached and floating cells were collected and lysed using 10% Pierce RIPA Buffer (Thermo Fisher Scientific). The cell lysate was resolved on a 10% Mini-PROTEAN TGX Precast Gel (Bio-Rad Laboratories Inc., Hercules, CA, USA), transferred onto polyvinylidene difluoride (PVDF) membranes, and blocked with SuperBlock Blocking Buffer (Thermo Fisher Scientific) for 1 h at room temperature of about 22 °C. Approximately 20–30 µg of total protein per lane was loaded for Western blot analysis. Although protein concentration was not directly quantified, these conditions reproducibly yielded clear signals within the linear range of detection in pilot experiments.

Membranes were incubated with primary antibodies against PD-L1 (1:1000), GAPDH (1:1000), Nectin-4/PVRL4 (1:1000), NF-κB p65 (1:1000), and STAT3 (1:1000) using the iBind Flex Western System (Thermo Fisher Scientific). Detection was performed using ECL Prime Western Blotting Detection Reagent (Global Life Sciences Technologies Japan, Tokyo, Japan), and images were captured with the ImageQuant LAS 4000 (Global Life Sciences Technologies Japan). Band intensities were quantified using ImageQuant TL (Global Life Sciences Technologies Japan), with GAPDH serving as the loading control for normalization.

### Real-time quantitative PCR for determining mRNA expression in RT4 cell lines

RT4 cells were seeded into 10 mL plates at a density of 5 × 10⁴ cells/well and incubated for 24 h. The cells were then treated with EV at 10 µg/mL for 6 h. Following treatment, mRNA expression levels were analyzed using real-time quantitative PCR (qPCR).

Total RNA was extracted using TRIzol RNA isolation reagent (Life Technologies, Carlsbad, CA, USA). First-strand cDNA synthesis was performed with the TaqMan Reverse Transcriptase Kit (Applied Biosystems, Branchburg, NJ, USA). Real-time qPCR was conducted on the StepOnePlus Real-Time PCR System (Applied Biosystems, Foster City, CA, USA).

Primers and probes were obtained from the Assays-on-Demand Gene Expression Assay (Applied Biosystems) for the following targets; (PD-L1 assay ID: CD274-Hs00204257_m1; STAST3 assay ID: STAT3-Hs00374280_m1; NFKB1 assay ID: Hs00765730_m1; GAPDH assay ID: Hs4326317E; B2M assay ID: Hs99999907_m1). Gene expression levels were determined using the comparative threshold cycle (Ct) method, with GAPDH or B2M serving as internal controls for normalization across samples.

### Statistical analysis

Data form Cell viability assay are expressed as mean ± standard error (SE). Statistical significance was defined as *p* < 0.05. Comparisons between groups were conducted using either the independent Student’s *t*-test or analysis of variance (ANOVA) for continuous variables. All statistical analyses were performed using SPSS for Windows (Version 25.0., IBM Corp., Armonk, NY, USA).

## Results

### EV treatment significantly reduced cell viability in UC cell lines

**Fig. 1:**
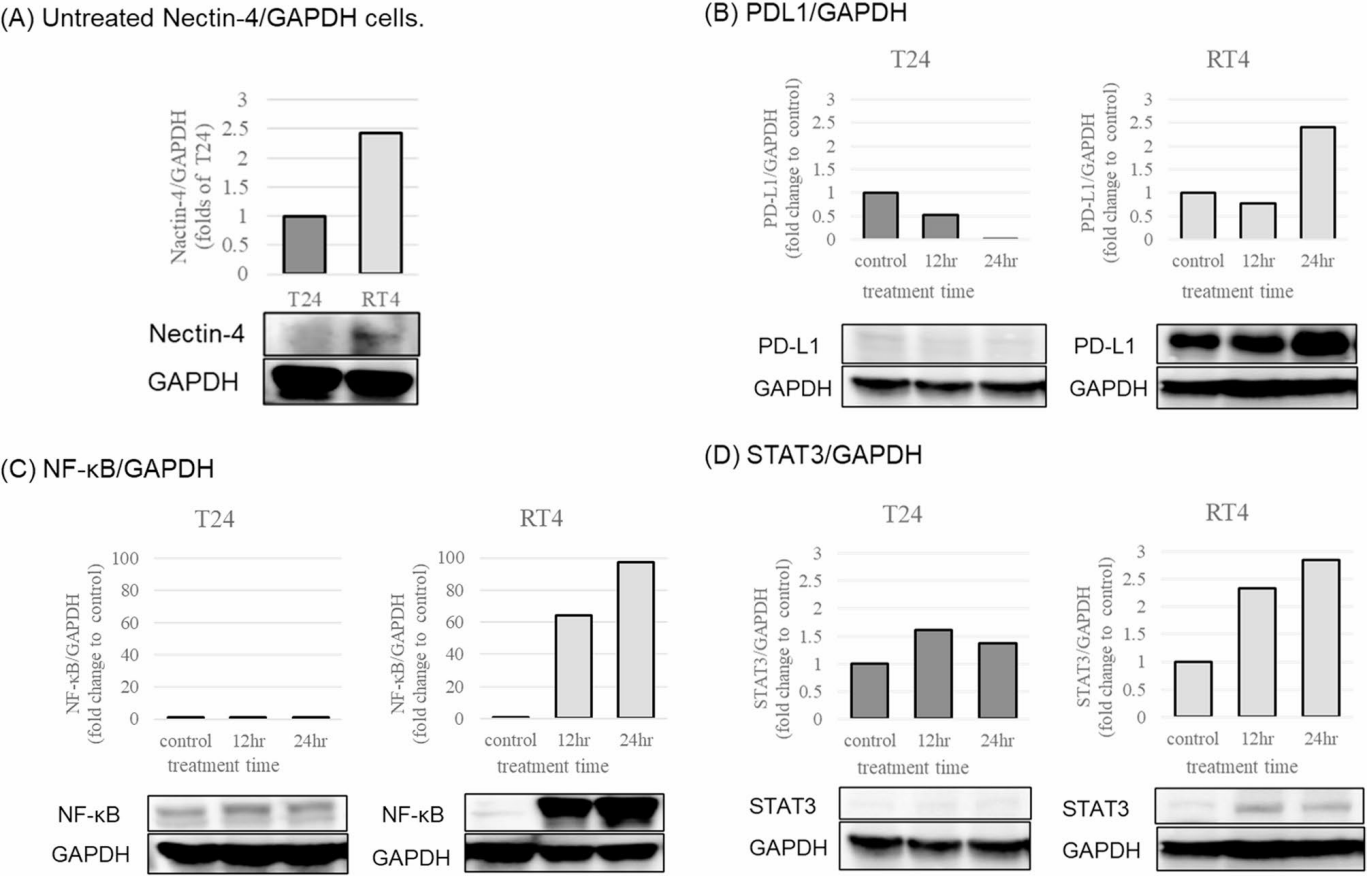
EV treatment significantly reduced the viability of UC cells. Mean ± standard error (SE) viability of RT4 and T24 cells treated with EV for 12–24 h and then re-incubated without EV for different durations, as determined using the MTT (3-(4,5-di-methylthiazol-2-yl)−2,5-diphenyltetrazolium bromide, yellow tetrazole) assay. Data represent mean ± SE from three independent experiments (*n* = 3). Analysis of variance with post-hoc Bonferroni test was used to compare the groups. Statistically significant (*p* < 0.05) decreases in cell viability compared with that of the control untreated cells are represented by asterisks (*)

EV treatment significantly reduced the cell viability in RT4 cells compared to that of the control group, irrespective of treatment duration or post-treatment incubation time. RT4 cells treated with 10 µg/mL EV exhibited approximately a 40% decrease in viability after 12 h treatment and a 60% decrease after 24 h treatment, following 12 h of post-treatment incubation. No changes in viability were observed with increasing durations of post-treatment incubation. Conversely, RT4 cells treated with 25 µg/mL EV showed a 50% reduction in viability after 12 h treatment and a 65% reduction after 24 h treatment, with viability declining further as post-treatment incubation increased (Fig. 1, A & B). In contrast, T24 cells treated with 10 µg/mL or 25 µg/mL EV for 12–24 h exhibited a significant decrease in viability following 12 h post-treatment incubation. However, cell viability gradually recovered, eventually returning to control levels with prolonged post-treatment incubation (Fig. 1, C & D).

### EV treatment induced PD-L1 expression in RT4 cells but not in T24 cells

**Fig. 2:**
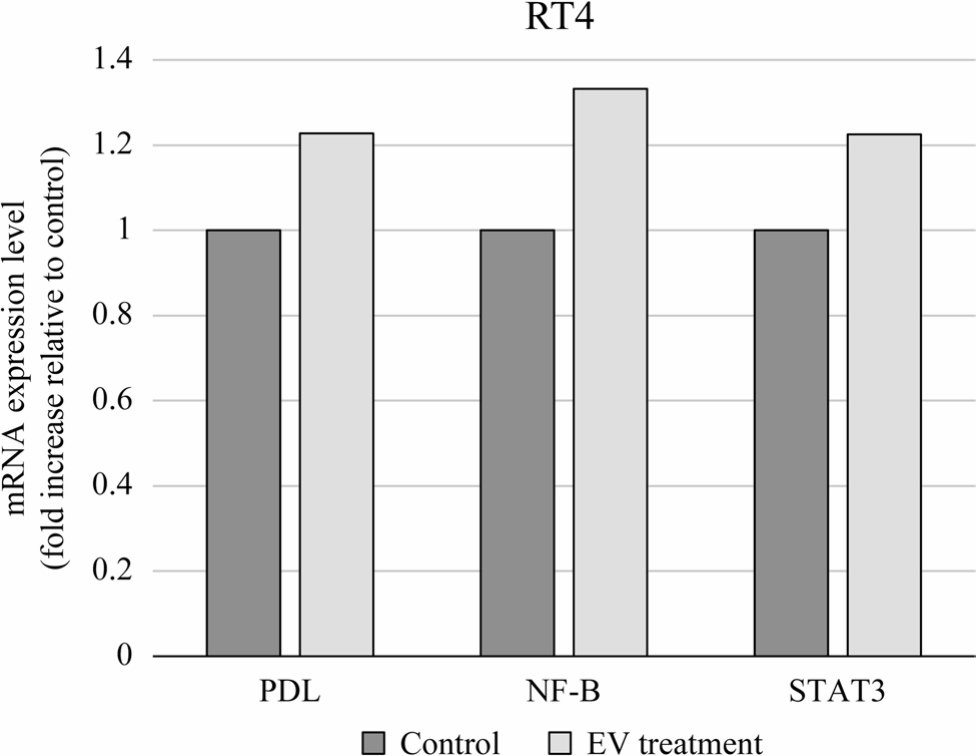
EV treatment induced PD-L1 expression in RT4 cells but not in T24 cells. **A** Representative western blot and relative band density of nectin-4 in RT4 and T24 cells. (**B**–**D**) Representative western blot and relative band density of (**B**) PD-L1, (**C**) NF-κB, and (**D**) STAT3 in RT4 and T24 cells treated with 10 µg/mL enfortumab vedotin (EV) for 12–24 h, evaluated after a 48-h post-treatment incubation period

Western blot analysis of untreated cells revealed that Nectin-4 expression in RT4 cells was 2.6-fold higher than that in T24 cells (Fig. 2A and Supplementary file). Following treatment with EV (10 µg/ml) for 12–24 h and a subsequent 48-h incubation, RT4 cells exhibited increased expression of PD-L1, NF-κB, and STAT3. In contrast, T24 cells displayed either diminished or only marginally increased expression of these proteins after EV treatment. The fold changes in relative band intensities, quantified using densitometry, are presented in Figs. 2B, C, D and Supplementary file. Specifically, the fold changes in PD-L1 expression were 0.761, 2.399, 0.517, and 0.006 for RT4/12 h, RT4/24 h, T24/12 h, and T24/24 h, respectively. For NF-κB, the values were 64.42, 97.03, 1.251, and 1.210, whereas STAT3 expression showed fold changes of 2.334, 2.844, 1.620, and 1.379 in the respective conditions.

### EV treatment enhanced the mRNA levels of PD-L1, NF-κB, and STAT3 in RT4 cell lines

**Fig. 3:**
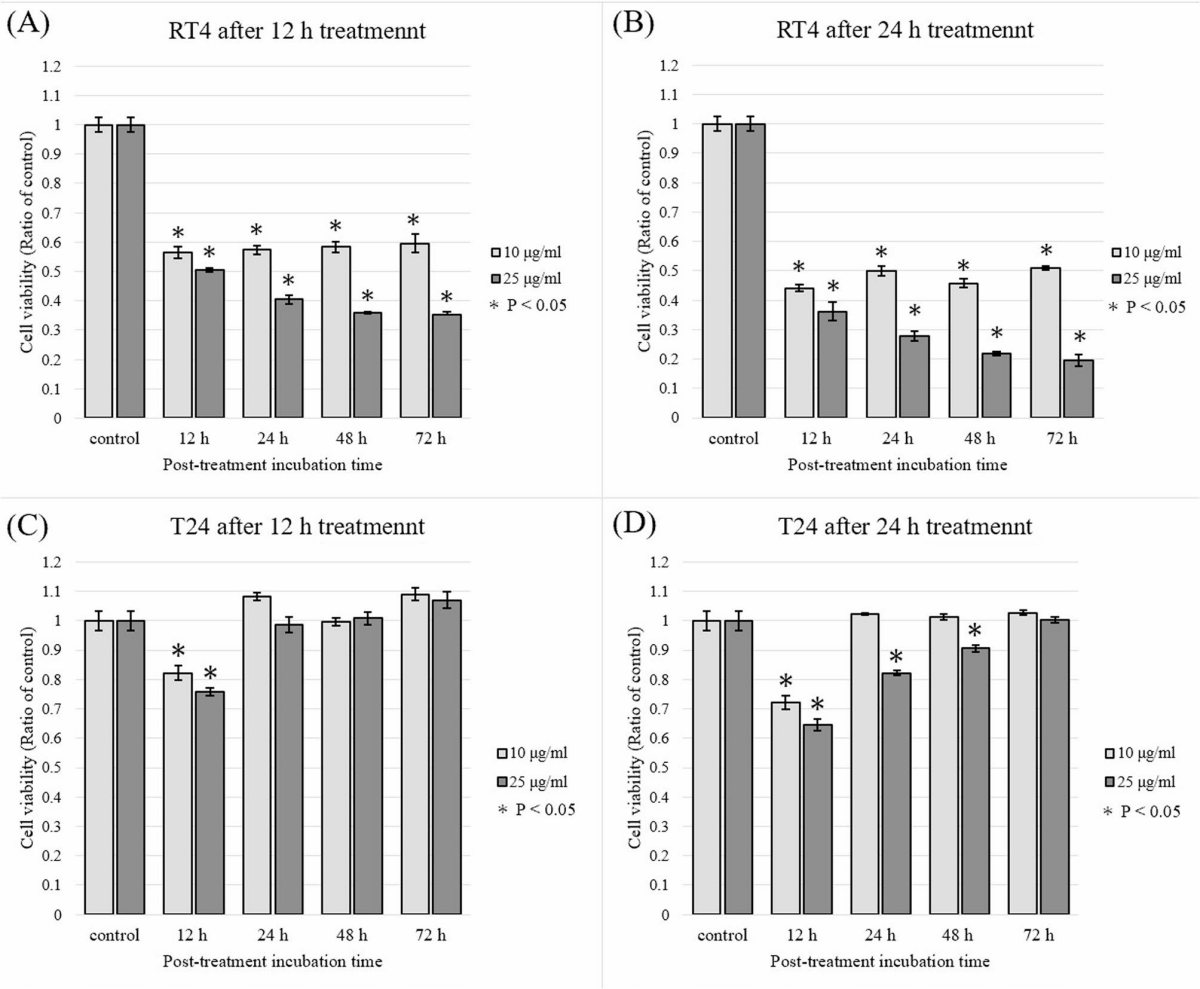
EV treatment upregulated the mRNA expression of PD-L1, NF-κB, and STAT3 in RT4 cells. Fold changes in the mRNA expression of PD-L1, NF-κB, and STAT3 in RT4 cells treated with 10 µg/mL enfortumab vedotin (EV) for 6 h compared with that in the un-treated controls. The data represent fold changes from a single experiment (*n* = 1) and are presented without statistical testing. mRNA expression was normalized to GAPDH using the comparative Ct method

To further investigate the molecular mechanisms underlying EV-induced PD-L1 expression, we assessed the mRNA levels of PD-L1, NF-κB, and STAT3 in RT4 cells following EV treatment. RT-qPCR analysis revealed that treatment with EV (10 µg/ml for 6 h) led to an increase in the transcriptional levels of these genes by 1.228-, 1.332-, and 1.225-fold for PD-L1, NF-κB, and STAT3, respectively, compared to untreated control cells. These results are based on a single experiment and are presented as fold change only, without statistical testing　(Fig. 3). These findings suggest that EV-mediated upregulation of PD-L1 is likely driven by the activation of NF-κB and STAT3 signaling pathways, reinforcing their roles in the regulatory mechanisms of immune checkpoint expression in RT4 cells.

## Discussion

PD-1/PD-L1 inhibitors have been established as effective treatments for locally advanced and metastatic UC, forming a standard therapeutic approach alongside systemic chemotherapy [5, 6]. Notably, UC cells with high PD-L1 expression have been associated with improved responses to PD-1/PD-L1 inhibitors [5, 6]. While previous studies have demonstrated that chemotherapy can induce PD-L1 expression [9–11], the impact of EV on PD-L1 regulation in UC remains unexplored. In this study, we provide the first evidence that EV, an antibody-drug conjugate targeting nectin-4, induces PD-L1 expression in a UC cell line expressing high levels of nectin-4. These findings suggest that combining EV with PD-1/PD-L1 inhibitors, either sequentially or concurrently, may enhance treatment efficacy in locally advanced and metastatic UC.

Western blot analysis revealed that untreated RT4 cells exhibited higher nectin-4 expression compared to T24 cells (Fig. 2A). A cell viability assay further demonstrated that EV exerted a stronger antitumor effect in nectin-4-high RT4 cells than in T24 cells (Fig. 1), indicating a correlation between nectin-4 expression and EV sensitivity. Additionally, analysis of cells that survived EV treatment revealed an increase in PD-L1 expression in RT4 cells (Figs. 2B and 3), suggesting that EV may upregulate PD-L1 as part of an adaptive cellular response.

PD-L1 expression is a well-established mechanism of immune evasion in cancer cells. Previous studies have identified that transcription factors NF-κB and STAT3 play a crucial role in inducing PD-L1 expression [12]. In addition to promoting PD-L1 expression, NF-κB and STAT3 contribute to immune escape and tumor progression by inhibiting apoptosis [12]. NF-κB directly regulates the PD-L1 promoter, whereas STAT3, activated through the MAPK/c-Jun pathway, can bind to the PD-L1 promoter and enhance NF-κB signaling [13, 14].

Activation of NF-κB has been implicated in chemotherapy resistance across multiple cancers, including gastric, pancreatic, and lung cancers, via the AKT-IκB pathway [15–18]. In this study, we observed that EV treatment triggered NF-κB activation in UC cell lines (Figs. 2C and 3). Moreover, paclitaxel, a microtubule inhibitor, has been shown to induce PD-L1 overexpression in ovarian cancer cells via an NF-κB-dependent mechanism [11]. Given that EV contains monomethyl auristatin E (MMAE), a microtubule-disrupting agent similar to paclitaxel, EV-induced PD-L1 upregulation likely follows a similar NF-κB-mediated pathway. This hypothesis is further supported by our findings of increased NF-κB expression at both the mRNA and protein levels in EV-treated UC cells.

STAT3 also plays a crucial role in PD-L1 upregulation across various malignancies [19–25]. The ATR/CHK1 pathway regulates PD-L1 expression by phosphorylating STAT3 following irradiation [23]. Additionally, STAT3 activation has been implicated in drug resistance. For instance, docetaxel-induced STAT3 activation promotes autophagy, contributing to chemotherapy resistance in castration-resistant prostate cancer [24]. Similarly, in lung cancer, afatinib-induced STAT3 activation reduces drug sensitivity, which can be reversed by inhibiting IL-6R/JAK1/STAT3 signaling [25]. In this study, we observed STAT3 activation in UC cell lines following EV treatment (Figs. 2D and 3), suggesting that EV may modulate PD-L1 expression via a STAT3-dependent pathway.

### Limitations and future directions

This study has several limitations. First, although we demonstrated that EV treatment is associated with increased PD-L1 expression in UC cell lines in vitro, PD-L1 regulation is influenced by the tumor immune microenvironment. Because this study lacks immune-competent or in vivo models, we cannot determine the functional impact of PD-L1 modulation. Future studies using animal models and clinical biospecimens will be required to assess the immunological relevance of these findings.

Second, while we observed EV-induced upregulation of NF-κB and STAT3, the precise molecular mechanisms remain unclear. Previous studies have demonstrated that chemotherapy activates NF-κB via the AKT-IκB pathway [15–18] and STAT3 via the IL-6R/JAK1 pathway, leading to PD-L1 expression and contributing to chemotherapy resistance in cancer cells [25]. Similar to these findings, our results suggest that PD-L1 upregulation in response to EV treatment may serve as an escape mechanism to its antitumor effects, potentially reducing treatment efficacy over time. Future studies evaluating the combination of EV with NF-κB and/or STAT3 inhibitors will be essential to elucidate the pathways involved in EV-induced PD-L1 expression and explore strategies to overcome resistance.

Third, the Western blot analysis of nectin-4 expression showed faint bands in T24 cells (Fig. 2A). While this may reflect the known lower expression of nectin-4 in basal-type bladder cancer cell lines such as T24 compared to luminal-type lines like RT4, as reported in previous studies [26, 27], the absence of a dedicated positive control and optimization steps limits the strength of this result. We have included unedited full-membrane images in the Supplementary file to enhance transparency. Future experiments using optimized detection conditions and positive control lysates will be necessary to validate this finding.

And the Western blot analysis of PD-L1 expression (Fig. 2B) was performed without biological replicates, and statistical comparison was not feasible. While densitometric quantification suggests a fold increase in PD-L1 expression, we acknowledge that the visual impression of the band intensity may not fully align with the calculated values. Additional biological replicates would strengthen the validity of this observation, but further experiments could not be performed due to limitations in reagent availability.

Fourth, only RT-qPCR for RT4 was performed, and data for RT-qPCR of T24 are missing. The reason we focused only on RT4 in the mRNA assay was that RT4 expressed high levels of nectin-4 in this study and was the cell line in which EV exposure most significantly regulated PD-L1 and signaling proteins at the protein level. On the other hand, T24 is generally reported in external resources as a basal-type cell line with low nectin-4 expression [26, 27]. We determined that RT4 is a more informative model for initial mRNA expression level analysis. In the future, validation of RT-qPCR in cell lines other than RT4 is necessary.

And, to further support the findings from the Western blot analysis (Fig. 2), we performed a single qRT-PCR experiment (Fig. 3), we acknowledge that the lack of biological replicates limits the statistical validation of these findings. While this limits the strength of the conclusion regarding transcriptional changes, the observed trend aligns with the protein expression data in Fig. 2, supporting the overall interpretation. Future studies with replicate experiments will be necessary to validate these findings.

Fifth, this study did not assess whether EV-induced PD-L1 expression is transient or sustained. Previous research has shown that paclitaxel transiently enhances PD-L1 expression via NF-κB activation in a mouse model of ovarian cancer [11]. Given that EV, similar to paclitaxel, is a microtubule inhibitor, a similar transient effect may occur. If PD-L1 expression is temporary, concurrent administration of EV and PD-1/PD-L1 inhibitors may be more effective than sequential therapy. Future studies should investigate the kinetics of PD-L1 expression following EV treatment to optimize treatment timing.

Recent clinical studies have shown that tumors with high membranous nectin-4 respond well to EV monotherapy [28], while the benefit of EV combined with PD-1/PD-L1 inhibitors appears to vary depending on molecular features [29]. In our study, EV treatment increased PD-L1 expression in the nectin-4-high RT4 cell line, but not in T24 cells with lower nectin-4. This suggests that nectin-4 expression may influence immune-related responses to EV. While our in vitro system does not model the immune microenvironment, these findings support a potential link between nectin-4 status and the effect of EV ± PD-1/PD-L1 inhibitors. Further clinical studies are needed to validate this hypothesis.

## Conclusion

This study provides the first evidence that EV is associated with increased PD-L1 expression, along with upregulation of NF-κB and STAT3, suggesting a mechanistic link that may contribute to immune modulation in nectin-4-high bladder cancer cells. These findings suggest that EV-induced PD-L1 upregulation could influence the efficacy of PD-1/PD-L1 inhibitors in UC. Future studies should explore the mechanistic pathways involved in this process and determine the clinical implications of combining EV with immunotherapy.

## Supplementary Information

Supplementary Material 1.

Supplementary Material 2.

## Data Availability

Data is provided within the manuscript, supplementary information files or related files.

## Abbreviations

UC: Urothelial carcinoma
EV: Enfortumab vedotin
PD-L1: Programmed death-ligand 1
NF-κB: nuclear factor-kappa B
STAT3: Signal Transducers and Activator of Transcription 3
MMAE: Monomethyl auristatin E
MTT: the 3-(4,5-dimethylthiazol-2-yl)-2,5-diphenyltetrazolium bromide
GAPDH: Glyceraldehyde-3-phosphate dehydrogenase
