# Supplementary figures and images for: Enfortumab vedotin promotes PD-L1 expression in urothelial carcinoma via NF-κB and STAT3 pathways highlighting mechanisms of immune evasion and potential for combination therapy
Source: BMC Immunol. 2025 Sep 25;26:70. doi: 10.1186/s12865-025-00751-2 (PMC12465518; doi:10.1186/s12865-025-00751-2)

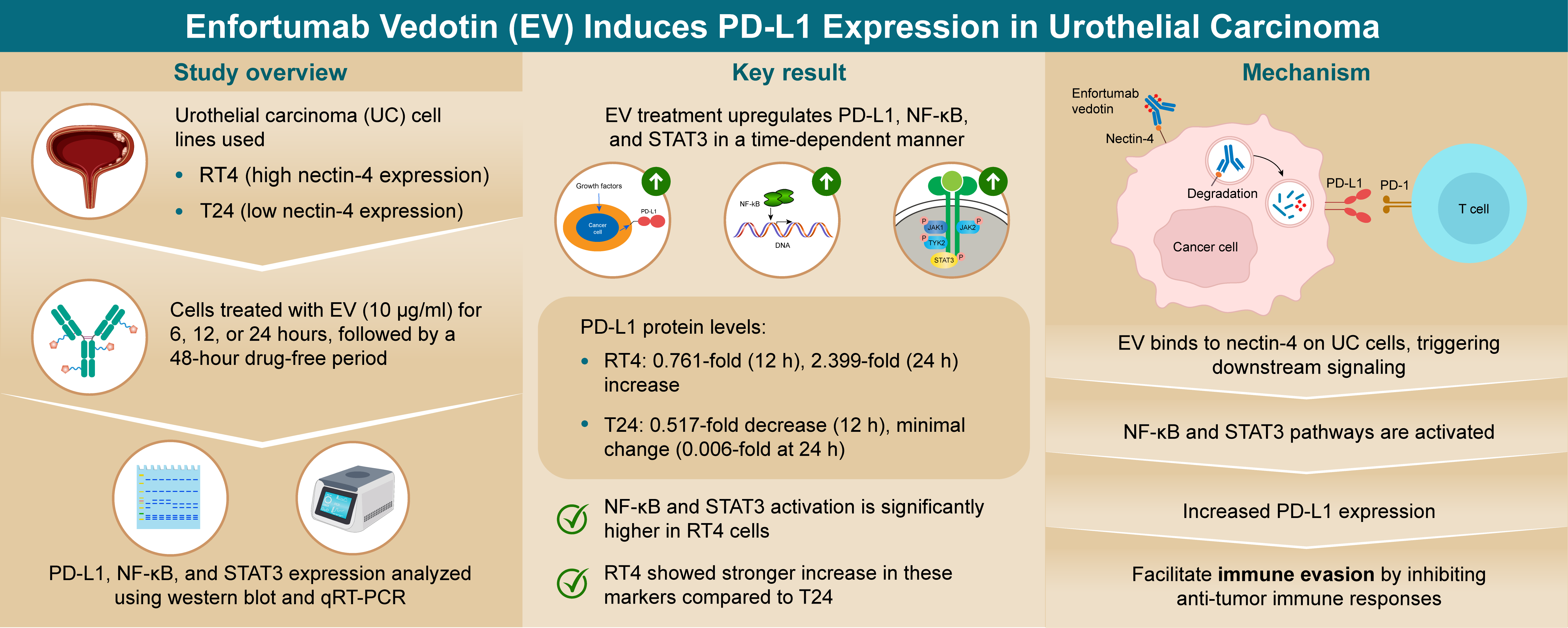

Supplement: Supplementary file 1 — Supplementary Material 1. [file 12865_2025_751_MOESM1_ESM.tif]
