# Supplementary material for: Enfortumab vedotin promotes PD-L1 expression in urothelial carcinoma via NF-κB and STAT3 pathways highlighting mechanisms of immune evasion and potential for combination therapy
Source: BMC Immunol. 2025 Sep 25;26:70. doi: 10.1186/s12865-025-00751-2 (PMC12465518; doi:10.1186/s12865-025-00751-2)

Supplementary file; Western blot images

These images are Western blot images of Figure2　A)　Untereated　Nectin-4/GAPDH.


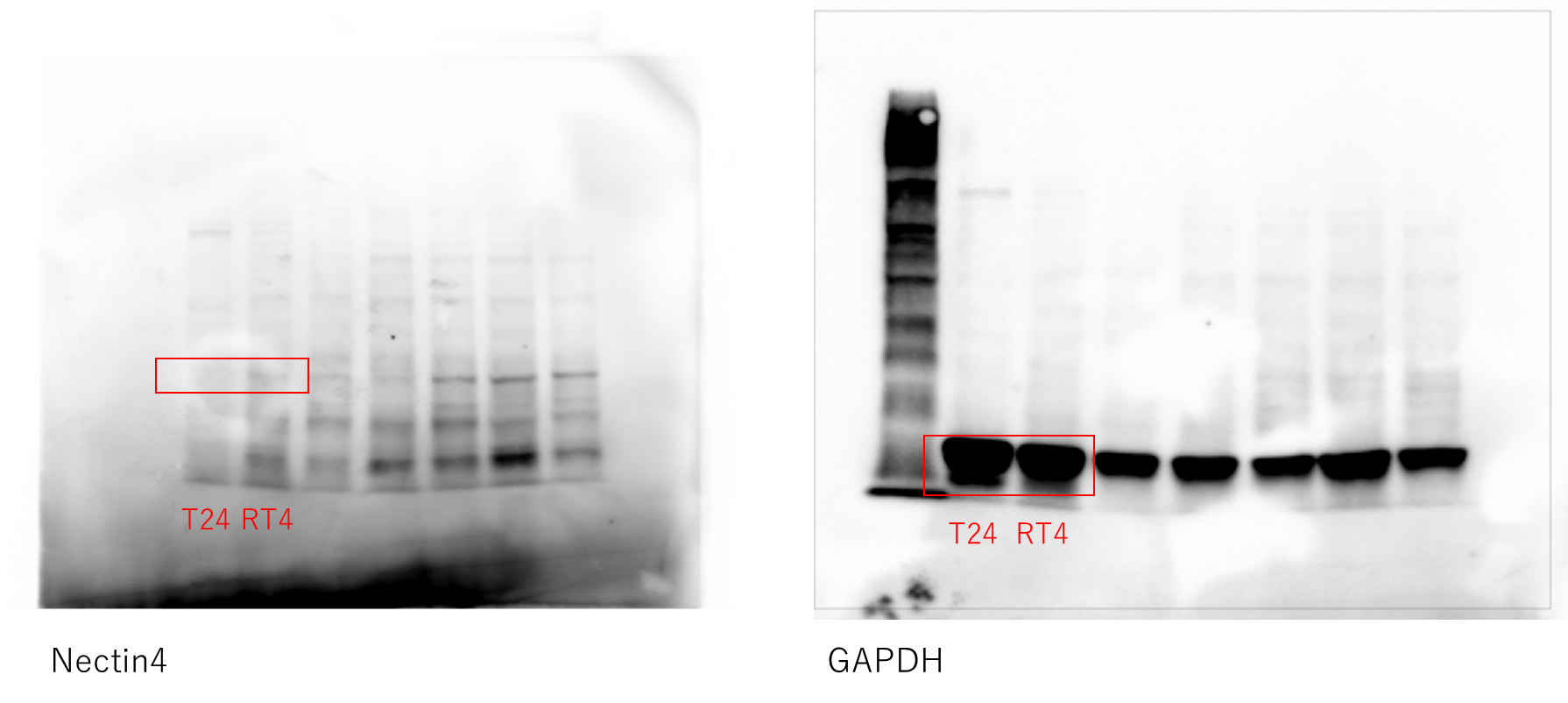
The area boxed in red are the image presented in the manuscript. Nectin-4 and GAPDH were evaluated on the same gel.

The images below are unprocessed. When Nectin was evaluated, the control ladder was not well expressed.

Nectin-4


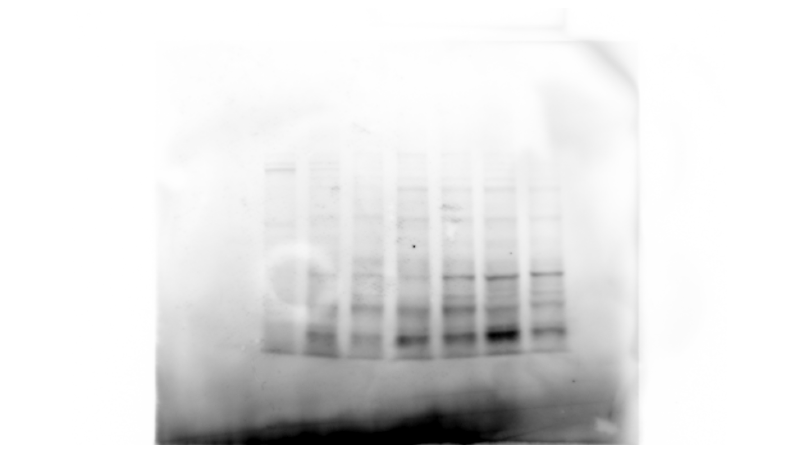


GAPDH

　
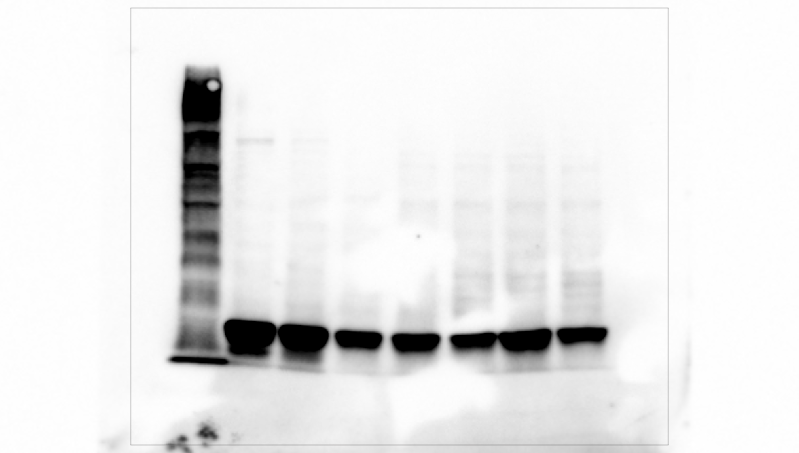


These images are Western blot images of Figure2 C) NFKB/GAPDH.

The area boxed in red are the image presented in the manuscript. NFKB and GAPDH were evaluated on the same gel.


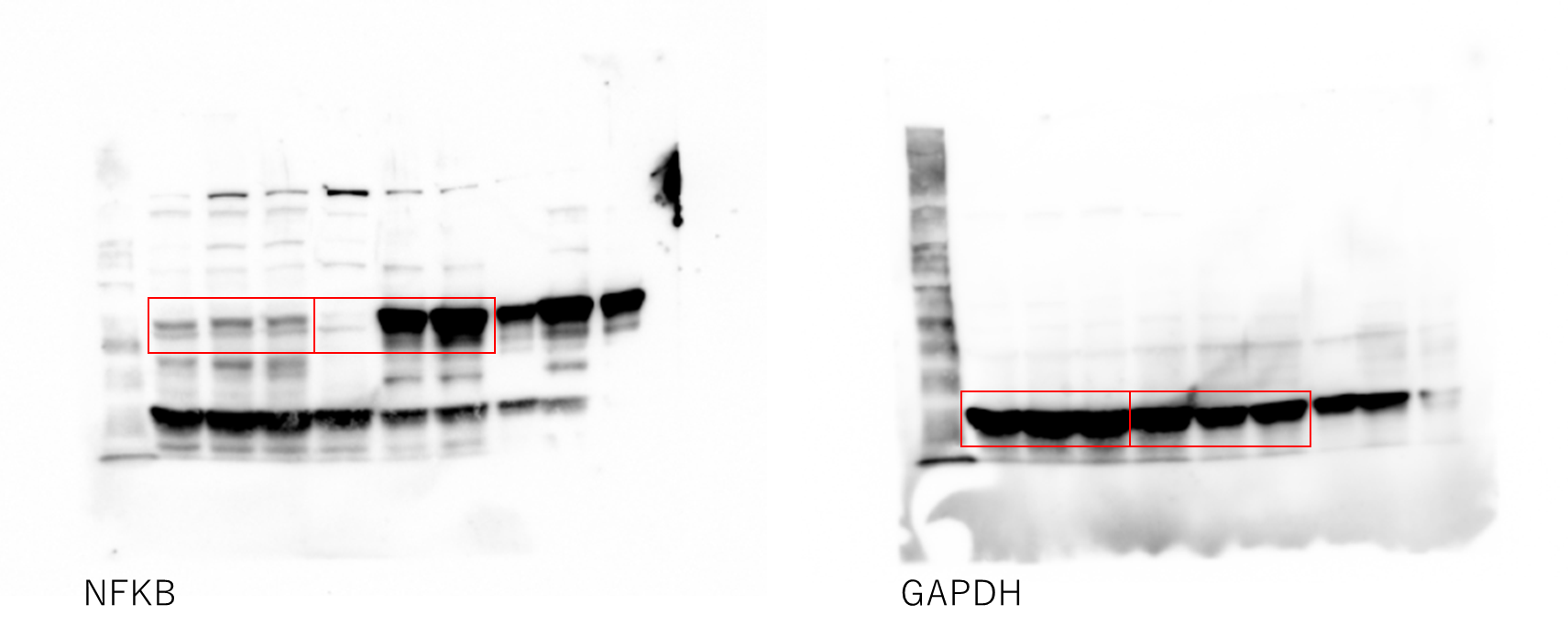


The images below are unprocessed.

NFKB


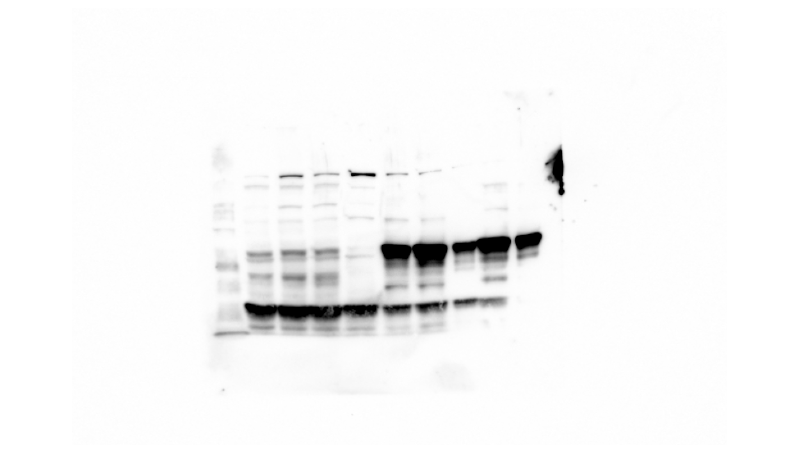


GAPDH


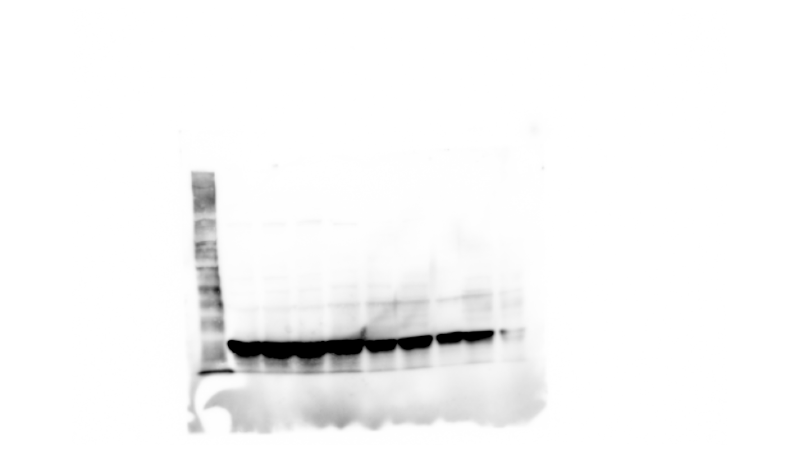


These images are Western blot images of Figure2 B) PD-L1/GAPDH and D) STAT3/GAPDH.


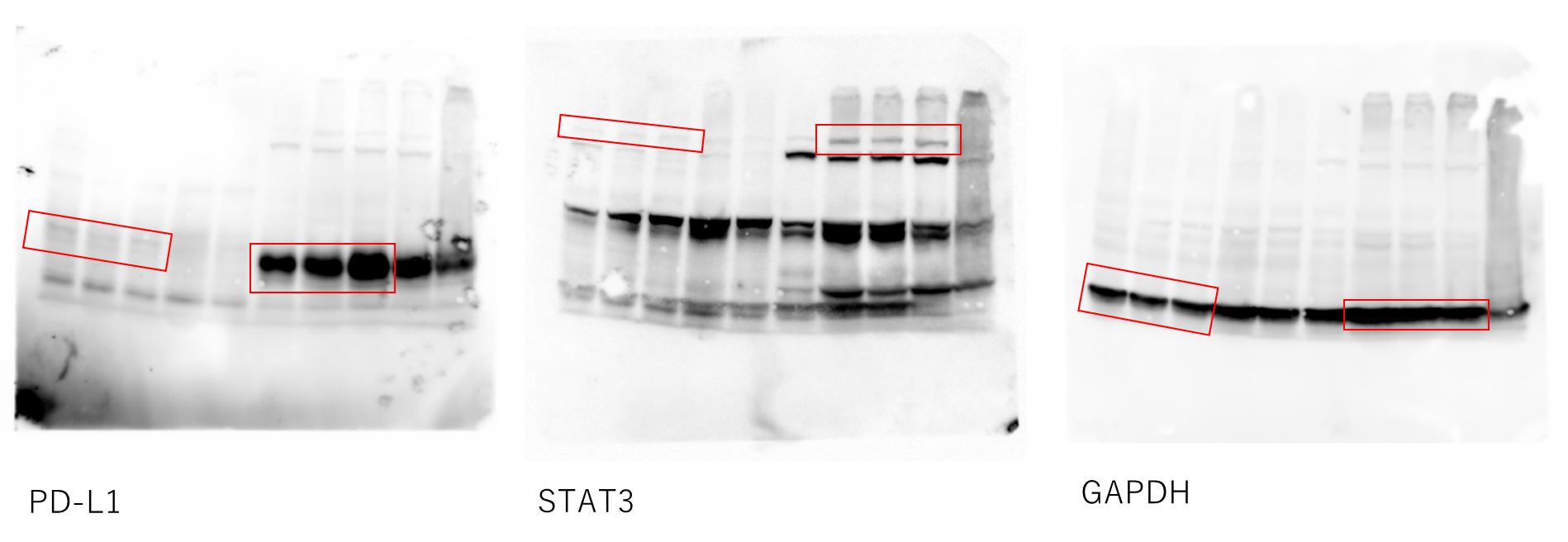
The area boxed in red is the image presented in the manuscript. PD-L1, STAT3, and GAPDH were evaluated on the same gel.

The images below are unprocessed.

PD-L1


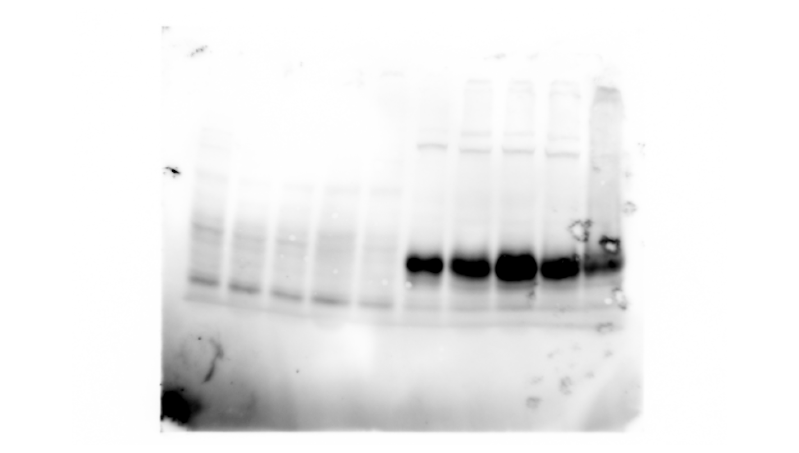


STAT3


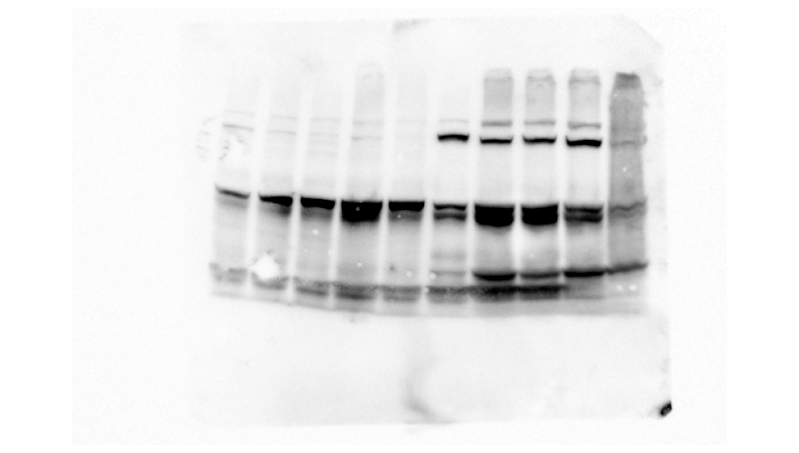


GAPDH


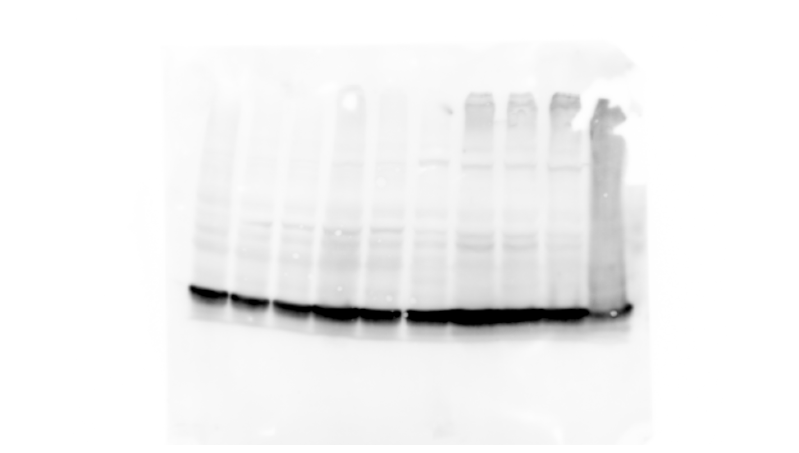

Supplement: Supplementary file 2 — Supplementary Material 2. [file 12865_2025_751_MOESM2_ESM.docx]
